# National Hospitals in the Valley of the Mississippi

**Published:** 1834

**Authors:** 


III.—ORIGINAL.

### The Western Journal.

*E Sylvis, acque atque ad Sylvas, Nuncius.*

Cincinnati, Ootober 1, 1834.

### I.—National Hospitals in the Valley of the Mississippi.

In a late number, we penned a few sentences on this subject, suggested by Dr. Campbell of St. Louis, and promised to resume it. The want of hospitals for the reception of those engaged in the laborious, unhealthy, and dangerous navigation of the Mississippi and its great tributary rivers, has long been felt. Impressed with this sentiment, the legislature of Louisiana, about the year 1819, proposed to the Mississippi states, to establish hospitals, in different places, and give to their citizens, in distress, reciprocal admission. But little, as far as we know, grew out of this suggestion. However, the legislature of Ohio, early in 1821, on the recommendation of Governor Brown, followed up by the personal application of the Editor of this Journal, instituted and endowed in this city, the existing charity, denominated the Commercial Hospital of Ohio; and some years afterwards the people of Louisville, aided by the state of Kentucky, established another in that place—provision of a similar kind having, mean while, been made in New Orleans. But these establishments, so far from supplying the wants of the great valley, have only served to render them more apparent. With the increase of population and commerce, spreading themselves up and down every stream that can float the humblest steam boat, the number of patients has been multiplied a thousand fold, since those hospitals were erected, and the points which call for similar establishments, increased in the same ratio. To comprehend the magnitude of our wants in this respect, we must glance at our actual condition.

The number of inhabitants, dependent more or less upon the Mississippi, as their great channel of communication with the ocean, is about 4,000,000. In descending the river with the produce and manufactures of western Pennsylvania and Virginia, Ohio, Indiana, Illinois, Missouri, Kentucky and Tennessee, to say nothing of Alabama, Mississippi, Arkansas and Louisiana, those engaged in the trade, go from a higher to a lower latitude; and as this commerce is chiefly carried on in the spring, early summer, and beginning of autumn, they are often exposed to great heat, and to all the causes of disease, which heat and moisture, acting on dead vegetable matter, can generate. The casualties of the river, moreover, are numerous and unavoidable—such as boats getting aground, and compelling the boatmen to expose themselves to "fire and water" in the rays of a burning sun and the current of the stream—the snagging and sinking of boats, and consequent lying out at night—the bursting of boilers, and aspersion of steam and hot water, over the crew and passengers—finally, the crash of boats against each other at night, by which the limbs and even lives of those who navigate them are often mutilated or destroyed. In these and other modes of smaller violence, a very great number of sick and disabled persons are constantly floating on our rivers, or left to perish (if nature should not save them) in cabins on the shore. Among these sufferers, are not a few who are persons of consequence to society, and have left respectable families, thus to perish on the river, or beneath the deep forests, which casts their gloomy shadows on the surface of its waters.

Such being the state of the interior, it is undeniable, that both humanity and patriotism call aloud for the establishment of houses of reception and relief, at so many different points, as to permit the sufferers from accident or disease, to be landed in due season, and treated with skill and kindness. We shall not undertake to designate where these hospitals should be placed, or even to suggest the number that are required, as this could only be done by a commission of experienced river traders and intelligent steam boat masters, with whom we shall leave the subject, and proceed to inquire by whom they should be erected.

The benevolent proposal of the state of Louisiana has not been successful; and, indeed, it cannot be expected that any public work, which requires the co-operation of eight or ten states, can be executed, except by the federal government. That government was instituted by the people of the states, expressly to perform such enterprises as would otherwise require the co-operation of several; and it is not, indeed, compatible with the integrity and quiet of the Union, for groups of states to go into special compacts. In this instance, it is true, no harm could come from such a subordinate confederacy, as whatever cements the Mississippi states more closely, tends to hold the Union together; but still it would be a violation of principle, and the general government should prevent such a step, by itself executing the work—we should rather say *national* work. That it would be as strictly and legitimately a national work, as any connected with the seaboard, and having for its object the promotion of the commerce of the country, will be admitted by all who may have the industry to examine it, and the candor to state the result of their inquiry.

We do not need fortifications, artificial harbors, light houses and breakwaters, in the interior, but we want commercial hospitals. Let it not be said, that the trade which renders them necessary, is merely a traffic between the people of the same state, or even of different states. It is a part of the great foreign commerce, exporting and importing, of the United States; and deserves to be fostered by the general government, not less than that which floats on the ocean. The relation between the interior and New Orleans, is not that of mere supply and consumption, but of supply and transmission. The produce of the most productive, if not quite the most extensive valley on earth, is concentrated at the mouth of the river, for shipment and dristribution over the Atlantic states, the West Indies, and the kingdoms of western Europe; and has already been started on the great voyage to distant lands, when it is embarked at Pittsburg, Cincinnati, Louisville, St. Louis, Nashville, or any of the hundred intermediate towns of lesser note.

But why do we confine our observations to the Valley of the Mississippi? The Basin of the Lakes has equal wants in this respect, and the whole should be embraced in one system. The coast, along which an active trade is already carried on, extends from near the eastern end of Lake Erie, to the western shore of Michigan—from Cleaveland to Chicago and Green Bay—embracing the northern frontier of Ohio, Michigan, Indiana and Illinois, and stretching out, when we follow its sinuosities, more than a thousand miles. As this coast is that of inland seas, which separate the United States from a foreign government, it is as much entitled to the fostering care of our own, as that which lies between Portland and Pensacola.

Believing as we do, that the constitution gives the power, we hold that it imposes the duty, of executing without delay, the public works to which we have referred; and we trust, that, as soon as an interlude in the angry drama of national politics may arrive, the federal government will admit this to be among the legitimate objects for which it was instituted, and seriously resolve upon its execution.

Mean while our readers in the great valley, (intelligent and numerous enough *of course* to exert a commanding influence on its representatives,) will, we trust, bestir themselves on this subject. It is undoubtedly the noblest work of patriotism and humanity, to which the profession of this region can direct their attention; and society has a right to expect from them an efficient exercise of their influence.

We would make a similar call on the editors of public journals in the west and south—indeed throughout the Union; for their assistance would be still more effective, than that of the medical profession. Those who publish in the Valley, will of course "lend a hand," and those who reside east of the mountains, could scarcely fail to co-operate, if they were duly to consider the matter; or even to take of it the limited and selfish view which would connect it merely with the interests of the people of the old states. The view to which we refer is this. A very great number of those who are engaged in the navigation and commerce of the Mississippi and its tributaries, are emigrants from the east, who have left friends, more favored by fortune, behind them, who ought not, who could not if they would, be indifferent to the perils and sufferings of their sons and brothers, that have sought to earn a subsistence in the land of promise—but which to many is a land of hard labor and exposure. Still further, the number of travellers, male and female, from the Atlantic states, that make voyages of business, observation, or pleasure upon our "western waters," is far greater than is generally known; and every year, many of them perish from casualties and disease, who might be preserved, if well conducted infirmaries were scattered throughout the great region, which they attempt to traverse.

